# Circulating activated protein C levels are not increased in septic patients treated with recombinant human soluble thrombomodulin

**DOI:** 10.1186/s12959-018-0178-0

**Published:** 2018-09-28

**Authors:** Takuro Arishima, Takashi Ito, Tomotsugu Yasuda, Nozomi Yashima, Hiroaki Furubeppu, Chinatsu Kamikokuryo, Takahiro Futatsuki, Yutaro Madokoro, Shotaro Miyamoto, Tomohiro Eguchi, Hiroyuki Haraura, Ikuro Maruyama, Yasuyuki Kakihana

**Affiliations:** 1 Emergency and Critical Care Center, Kagoshima University Hospital, Kagoshima, Japan; 2 Department of Systems Biology in Thromboregulation, Kagoshima University Graduate School of Medical and Dental Sciences, 8-35-1 Sakuragaoka, Kagoshima, 890-8544 Japan; 3 Emergency and Intensive Care Medicine, Kagoshima University Graduate School of Medical and Dental Sciences, Kagoshima, Japan

**Keywords:** Disseminated intravascular coagulation, Protein C, Sepsis, Thrombomodulin

## Abstract

**Background:**

Recombinant human soluble thrombomodulin (rTM) has been used for the treatment of disseminated intravascular coagulation in Japan, and an international phase III clinical trial for rTM is currently in progress. rTM mainly exerts its anticoagulant effects through an activated protein C (APC)-dependent mechanism, but the circulating APC levels after rTM treatment have not been clarified. This prospective observational study investigated plasma APC levels after rTM treatment.

**Methods:**

Plasma levels of soluble thrombomodulin, thrombin-antithrombin complex (TAT), protein C, and APC were measured in eight septic patients treated with rTM. APC generation in vitro was assessed in the presence or absence of rTM.

**Results:**

rTM significantly increased thrombin-mediated APC generation in vitro. In septic patients, soluble thrombomodulin levels were significantly increased during a 30–60-min period of rTM treatment and TAT levels were decreased. However, APC activity was not increased during the treatment period.

**Conclusions:**

Plasma APC activity is not increased in septic patients treated with rTM. It is possible that APC acts locally and does not circulate systemically.

**Electronic supplementary material:**

The online version of this article (10.1186/s12959-018-0178-0) contains supplementary material, which is available to authorized users.

## Background

Thrombomodulin is an anticoagulant protein expressed on the surface of endothelial cells [1]. Thrombomodulin binds to thrombin and boosts its potential for activating protein C. Activated protein C (APC) then inactivates coagulation factors Va and VIIIa, limiting the amplification of blood coagulation [2]. In septic conditions, thrombomodulin expression can be compromised, leading to sepsis-associated disseminated intravascular coagulation (DIC) [3]. Substitution with recombinant human soluble thrombomodulin (rTM) is a therapeutic option in Japan under these conditions [4, 5], and an international phase III clinical trial for rTM is currently in progress.

Recombinant human APC (rhAPC) originally showed a significant reduction in all-cause mortality in patients with severe sepsis [6]. However, the initial success was not replicated in subsequent clinical trials involving adult patients with severe sepsis and low risk of death [7], children with severe sepsis [8], and patients with septic shock [9]. As a result, rhAPC has been withdrawn from the market. Although rTM shares its fundamental mechanism of action with rhAPC, it has several unique features. For example, rTM did not increase the incidence of bleeding complications [10], unlike the case for rhAPC [11]. This may be because rTM preferentially exerts its APC-dependent anticoagulant effects when and where thrombin exists [1]. In this context, it is important to consider whether APC acts locally or circulates systemically after rTM treatment. However, there has been no evidence for plasma APC levels after rTM treatment.

Recently, a novel method for measurement of plasma APC levels has been developed [12]. The method employs a single-stranded DNA aptamer that captures APC with high affinity and high specificity without capturing inactive protein C. Using this aptamer-based enzyme capture assay, we examined the plasma APC levels in septic patients treated with rTM. We found that circulating APC levels were not increased in these patients even though soluble TM levels were significantly increased by rTM treatment.

## Methods

### In vitro APC generation assay

Pooled normal plasma (George King Bio-Medical, Overland Park, KS) was incubated with thrombin (Mochida Pharmaceutical, Tokyo, Japan) and rTM (Asahi Kasei Pharma, Tokyo, Japan) at 37 °C for 10 min. The reaction was terminated by addition of a protease inhibitor cocktail containing hirudin and aprotinin (Sekisui Medical, Tokyo, Japan). In some experiments, protein C deficient plasma (Affinity Biologicals, Ancaster, Canada), antithrombin deficient plasma (Affinity Biologicals), protein C (kindly provided by Chemo-Sero-Therapeutic Research Institute, Kumamoto, Japan), and antithrombin (Japan Blood Products Organization, Tokyo, Japan) were used for preparing plasma with various concentrations of protein C and antithrombin. For pre-analytical APC stability assays, whole blood samples from three healthy volunteers were spiked with 16.7 ng/mL equivalent activity of APC (Haematologic Technologies, Essex Junction, VT) followed by addition of the protease inhibitor cocktail. Samples were stored at − 80 °C until analysis of APC concentrations.

### Patients and blood sampling

This prospective observational study conformed to the provisions of the Declaration of Helsinki and was approved by the Ethics Committee of Kagoshima University Hospital. Between May 2016 and March 2017, written informed consent was obtained from eight patients with sepsis-associated DIC prior to participation in the study. Although the number of enrolled patients did not reach the recruiting goal of this study, we decided not to extend the study period because the data of eight patients were enough to support the conclusion that plasma APC activity was not significantly increased in septic patients treated with rTM. Diagnoses of sepsis and DIC were made according to the Third International Consensus Definition for Sepsis (Sepsis-3) [13] and the diagnostic criteria established by the Japanese Association for Acute Medicine (JAAM DIC criteria) [14], respectively. Blood samples were collected from intravascular catheter in eight patients with sepsis-associated DIC before and after administration of rTM (130 or 380 U/kg, intravenous drip infusion; Asahi Kasei Pharma, Tokyo, Japan) on day 1 and day 2. The samples were immediately anticoagulated with one-tenth volume of sodium citrate and kept at 4 °C to minimize covalent inhibition of APC by plasma proteins. The samples were then centrifuged at 2000×*g* for 10 min at 4 °C. Part of the citrated plasma was mixed with the protease inhibitor cocktail containing hirudin and aprotinin within 15 min after blood sampling and stored at − 80 °C until analysis of APC concentrations. APC measurement with this protocol was comparable to the authentic protocol [12] in which aprotinin was added to blood collection tubes prior to blood sampling (data not shown).

### Measurement of plasma levels of APC, soluble thrombomodulin (sTM), protein C, thrombin-antithrombin complex (TAT), and prothrombin fragment 1 + 2 (F1 + 2)

Plasma APC levels were analyzed by the OLIGOBIND APC activity assay (Sekisui Diagnostics GmbH, Pfungstadt, Germany) according to the manufacturer’s instructions. Plasma sTM levels were measured by ELISA using monoclonal antibodies against thrombomodulin. The plasma sTM levels determined by this technique were well correlated with a functional assay [15]. Plasma protein C levels were measured by a synthetic chromogenic substrate method using HemosIL Protein C (Instrumentation Laboratory, Bedford, MA). Plasma TAT levels were analyzed using STACIA CLEIA TAT (LSI Medience, Tokyo, Japan) according to the manufacturer’s instructions. Plasma F1 + 2 levels were analyzed using Enzygnost F1 + 2 (Siemens Healthcare Diagnostics, Tokyo, Japan) according to the manufacturer’s instructions.

### Statistical analysis

In vitro APC generation assays were evaluated by analysis of variance followed by a Tukey–Kramer test. For assessment of clinical samples, a paired *t*-test was used for comparisons before and after rTM treatment. Data were presented as mean ± SD. A probability of < 0.05 was considered significant.

## Results and discussion

**Fig. 1:**
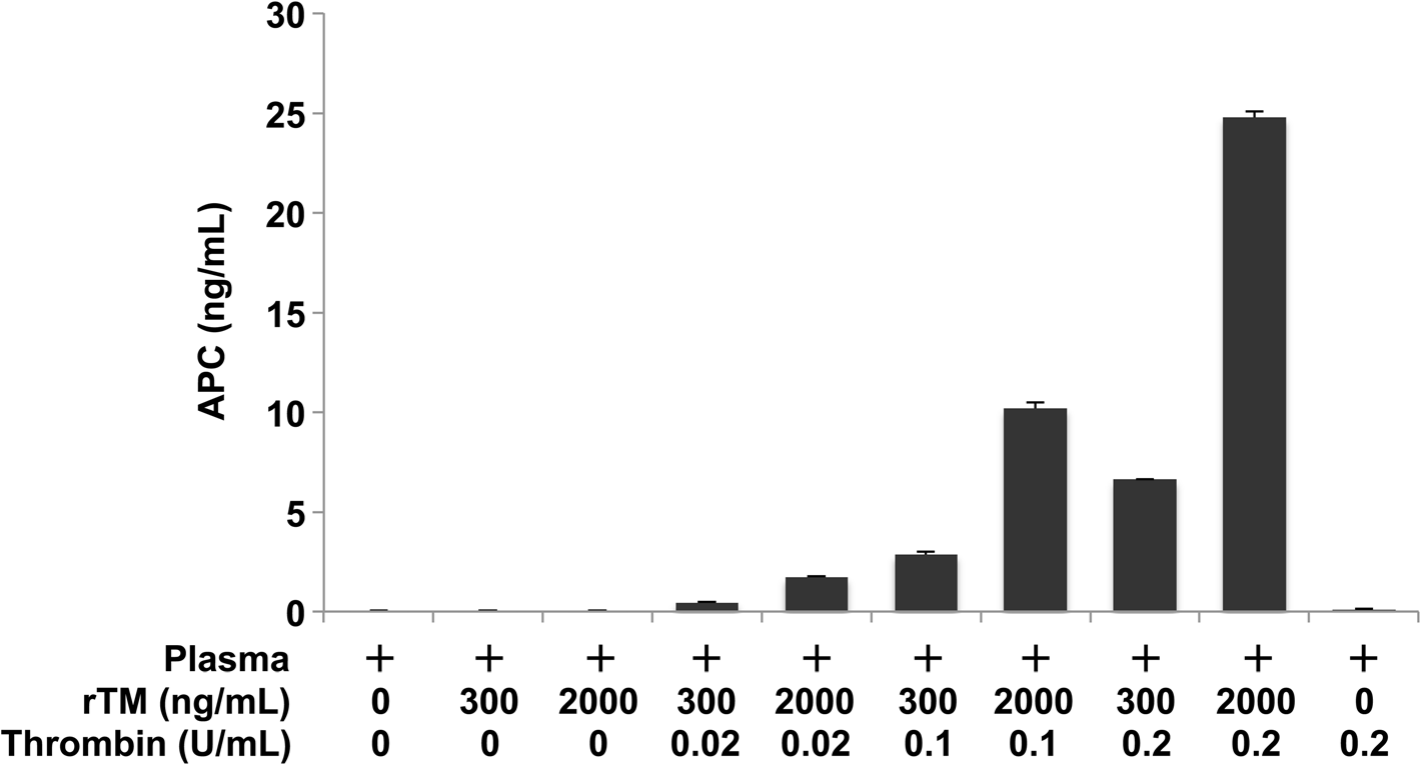
rTM promotes thrombin-mediated APC generation in vitro. Pooled normal plasma was incubated with thrombin (0–0.2 U/mL) and rTM (0–2000 ng/mL) at 37 °C for 10 min. APC levels were analyzed by the OLIGOBIND APC activity assay. The data represent mean ± SD (*n* = 3). Representative data of two independent experiments are shown

First, we conducted in vitro APC generation and quantification assays to verify that rTM can promote thrombin-mediated APC generation and that the aptamer-based enzyme capture assay can detect APC quantitatively. As shown in Fig. 1, thrombin alone or rTM alone did not induce APC generation. In contrast, the combination of thrombin and rTM increased APC generation in a dose-dependent manner. The concentrations of rTM in these assays were equivalent to those detected in our clinical samples (Fig. 3a). TAT levels in our in vitro samples (Additional file 1: Figure S1) were also equivalent to those in our clinical samples (Fig. 3b), suggesting that thrombin concentrations used in our in vitro assays were reasonable. These results indicate that rTM can promote thrombin-mediated APC generation in vitro, and that the assay can detect this generation.

**Fig. 2:**
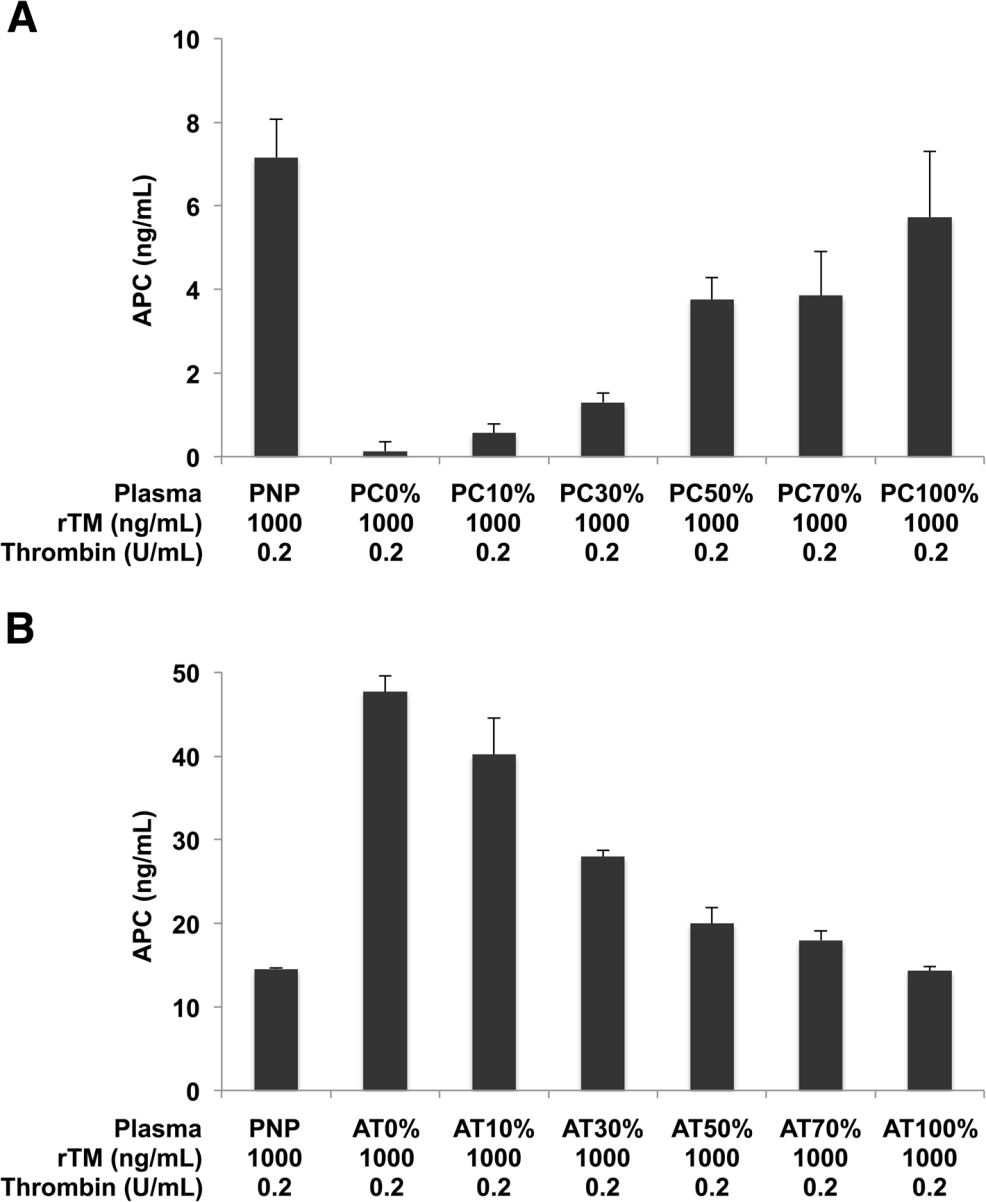
Decreased concentrations of protein C and antithrombin are associated with decreased and increased APC generation, respectively. **a** Plasma samples with 0, 10, 30, 50, 70, and 100% protein C levels were prepared by the addition of vehicle, 0.36, 1.08, 1.81, 2.53, and 3.61 μg/mL of protein C, respectively, to protein C deficient plasma. **b** Plasma samples with 0, 10, 30, 50, 70, and 100% antithrombin levels were prepared by the addition of vehicle, 0.07, 0.25, 0.43, 0.61, and 0.88 U/mL of antithrombin, respectively, to antithrombin deficient plasma. These plasma samples were incubated with 0.2 U/mL of thrombin (T1063, Sigma-Aldrich, St. Louis, MO) and 1000 ng/mL of rTM at 37 °C for 10 min. APC levels were analyzed by the OLIGOBIND APC activity assay. The data represent mean ± SD (n = 3). Representative data of two independent experiments are shown. PNP: pooled normal plasma, PC: protein C, AT: antithrombin

Next, we examined the impact of protein C and antithrombin levels on APC generation in vitro. Protein C and antithrombin are a substrate and an inhibitor of thrombin, respectively, and these levels are expected to have significant impacts on thrombin-mediated APC generation. As expected, APC generation was decreased as plasma protein C levels decreased (Fig. 2a). In contrast, APC generation was increased as plasma antithrombin levels decreased (Fig. 2b). These findings indicate that APC generation can be increased or decreased depending on the balance of antithrombin and protein C levels.

**Fig. 3:**
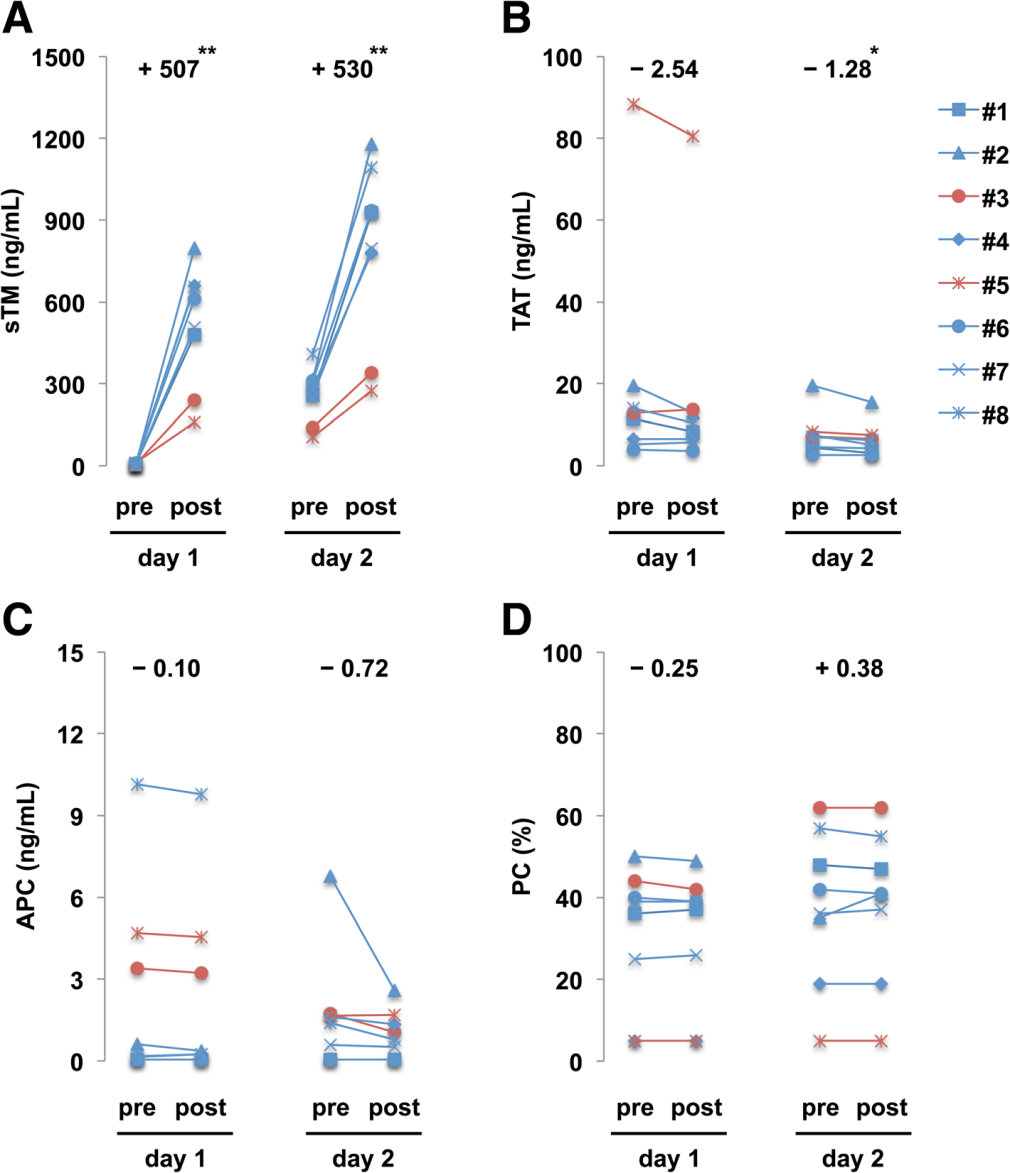
Plasma APC levels are not increased in septic patients during a 30–60-min period of rTM treatment, while TAT levels are decreased during the treatment period. **a** Blood samples were collected from eight patients with sepsis-associated DIC before and after administration of rTM (130 U/kg: red symbols; 380 U/kg: blue symbols) on day 1 and day 2. Plasma sTM levels were determined by ELISA. **b** Plasma TAT levels were analyzed by STACIA CLEIA TAT. **c** Plasma APC levels were analyzed by the OLIGOBIND APC activity assay. **d** Plasma protein C levels were measured by a synthetic chromogenic substrate method using HemosIL Protein C. A paired *t*-test was used for comparisons before and after rTM treatment. *P < 0.05, **P < 0.01

Then, we examined the plasma levels of sTM, TAT, protein C, and APC in patients with sepsis-associated DIC before and after administration of rTM. The background characteristics of the patients are summarized in Table 1. Plasma sTM levels rapidly increased during a 30–60-min period of rTM treatment on day 1 and day 2 (Fig. 3a). The detected sTM levels were essentially equivalent to those reported in previous clinical studies [16, 17] and those adopted in our in vitro assays. Plasma TAT levels were slightly, but significantly, decreased during a 30–60-min period of rTM treatment on day 2 (Fig. 3b). Plasma F1 + 2 levels remained unchanged during this treatment period (day 1 pre 557 ± 418 vs day 1 post 553 ± 415, day 2 pre 403 ± 340 vs day 2 post 395 ± 325). Plasma APC levels were not increased after rTM treatment, and instead showed a declining trend (Fig. 3c). Plasma protein C levels remained unchanged during this treatment period (Fig. 3d). These findings indicate that rTM promotes thrombin-mediated APC generation in vitro, but does not increase plasma APC levels in patients with sepsis-associated DIC.

**Table 1:** Background characteristics of the patients with sepsis-associated DIC

Case	1	2	3	4	5	6	7	8
Age	69	39	72	65	64	71	67	81
Sex	F	M	M	M	M	M	M	M
Site of infection	Abd	Pulm	Pulm	Abd	Abd	Pulm	Abd	Pulm
APACHE II	7	13	21	36	28	23	30	22
SOFA	5	7	11	13	18	12	13	9
SIRS	4	3	3	3	4	3	4	2
DIC score	5	8	5	4	8	8	5	5
AT activity	78	80	58	44	33	74	55	77
Concomitant anticoagulants		NM	AT NM	AT NM	AT NM	AT	AT NM	NM
Outcome	Alive	Alive	Dead	Alive	Dead	Alive	Alive	Dead

*F* female, *M* male, *Abd* abdominal, *Pulm* pulmonary, *APACHE* acute physiology and chronic health evaluation, *SOFA* sequential organ failure assessment, *SIRS* systemic inflammatory response syndrome, *DIC* disseminated intravascular coagulation, *AT* antithrombin, *NM* nafamostat mesilate

APACHE II score was evaluated on the first day of intensive care unit admission. Overall prognosis was evaluated on day 28 after rTM treatment. All other parameters, including SIRS score, SOFA score, DIC score, and AT activity, were evaluated on the first day of rTM treatment

It remains a matter of debate why rTM treatment did not increase plasma APC levels in septic patients. The anticoagulant effects of rTM treatment have been demonstrated in ex vivo settings where prothrombinase activity was attenuated in plasma containing rTM when compared to plasma without rTM [15, 18]. The anticoagulant property of rTM has also been demonstrated in clinical settings where TAT, D-dimer, or fibrin/fibrinogen degradation products (FDP) concentrations were lower in the rTM treatment group compared with the control group [10, 19, 20]. Our results also showed a decrease in the TAT concentration after rTM treatment (Fig. 3b). The anticoagulant effects of rTM appear to be mainly mediated by APC, because subjects with factor V Leiden, an APC-resistant factor V variant, showed less pronounced anticoagulant effects after rTM treatment [15]. These findings indicate that the anticoagulant effects of rTM are evident in clinical settings and appear to be APC-dependent.

There could be some possible explanations for why deliverable APC was not detected in the plasma of septic patients after rTM treatment. One possibility is that APC acted locally and did not circulate systemically. APC conveys its anticoagulant activity when bound to phospholipids in the plasma membrane of activated platelets or other types of cells. APC also exerts multiple cytoprotective effects when bound to endothelial protein C receptor. Furthermore, APC in the systemic circulation can be inactivated by plasma proteins, such as protein C inhibitor, antitrypsin, and α2-macroglobulin. Thus, APC mainly acts on the surface of platelets and endothelial cells in focal coagulation sites, and a large part of APC generated in vivo may not circulate in the active form. The second possibility is that protein C levels and thrombin levels in septic patients were insufficient for APC generation. Plasma protein C levels can be decreased in septic patients in part due to decreased production of protein C in the liver and increased leakage to the extravascular space. Thrombin generation can be decreased in patients treated with rTM due to APC-mediated inactivation of the coagulation cascade. It is possible that decreased protein C and thrombin may blunt APC generation in septic patients treated with rTM. The third possibility is that plasma APC levels might be increased at other time points. rTM was administered to patients via drip infusion over a period of 30–60 min. Plasma rTM concentrations peak immediately after the completion of rTM administration [16]. In our in vitro APC generation assays, APC generation peaked within 10 min and the activity of APC was decreased over time probably because protein C inhibitor, antitrypsin, and α2-macroglobulin bound to and inactivated APC. Based on these findings, blood samples were collected immediately after the completion of rTM administration in this study. However, it is possible that APC generation in vivo requires more time, especially when APC is generated in the extravascular space. The fourth possibility is that APC was inactivated during handling of plasma samples. This is unlikely because all plasma samples were mixed with a protease inhibitor cocktail within 15 min after blood sampling and reconstituted APC was stable during this period (Additional file 2: Figure S2). Taken together, the present findings suggest that APC does not circulate systemically in the active form after rTM treatment. This may explain why bleeding complications occur infrequently after rTM treatment. However, we cannot completely exclude the possibility that rTM exerts anticoagulant effects in an APC-independent manner.

## Conclusions

Plasma APC activity is not increased in septic patients treated with rTM. It is possible that APC acts locally and does not circulate systemically.

## Additional files

Additional file 1: **Figure S1.** TAT levels in in vitro APC generation assays. Pooled normal plasma was incubated with thrombin T1063 (0–0.2 U/mL) and rTM (0–2000 ng/mL) at 37 °C for 10 min. TAT levels were analyzed by STACIA CLEIA TAT. The data represent mean ± SD. Representative data of two independent experiments are shown. (TIF 547 kb)

Additional file 2: **Figure S2.** Reconstituted APC is stable at 4 °C for 15 min before adding the protease inhibitor cocktail. For pre-analytical APC stability assays, whole blood samples from three healthy volunteers were spiked with 16.7 ng/mL equivalent activity of APC and kept at 4 °C. The protease inhibitor cocktail (PI cocktail) was added either before or 15 min after the APC spike. Plasma APC levels were analyzed by the OLIGOBIND APC activity assay. The data represent mean ± SD. Representative data of two independent experiments are shown. (TIF 640 kb)

## Abbreviations

APACHE: Acute physiology and chronic health evaluation
APC: Activated protein C
DIC: Disseminated intravascular coagulation
ELISA: Enzyme-linked immunosorbent assay
F1 + 2: Prothrombin fragment 1 + 2
FDP: Fibrin/fibrinogen degradation products
rhAPC: Recombinant human activated protein C
rTM: Recombinant human soluble thrombomodulin
SIRS: Systemic inflammatory response syndrome
SOFA: Sequential organ failure assessment
sTM: Soluble thrombomodulin
TAT: Thrombin-antithrombin complex
