# Supplementary figures and images for: Circulating activated protein C levels are not increased in septic patients treated with recombinant human soluble thrombomodulin
Source: Thromb J. 2018 Sep 28;16:24. doi: 10.1186/s12959-018-0178-0 (PMC6161343; doi:10.1186/s12959-018-0178-0)

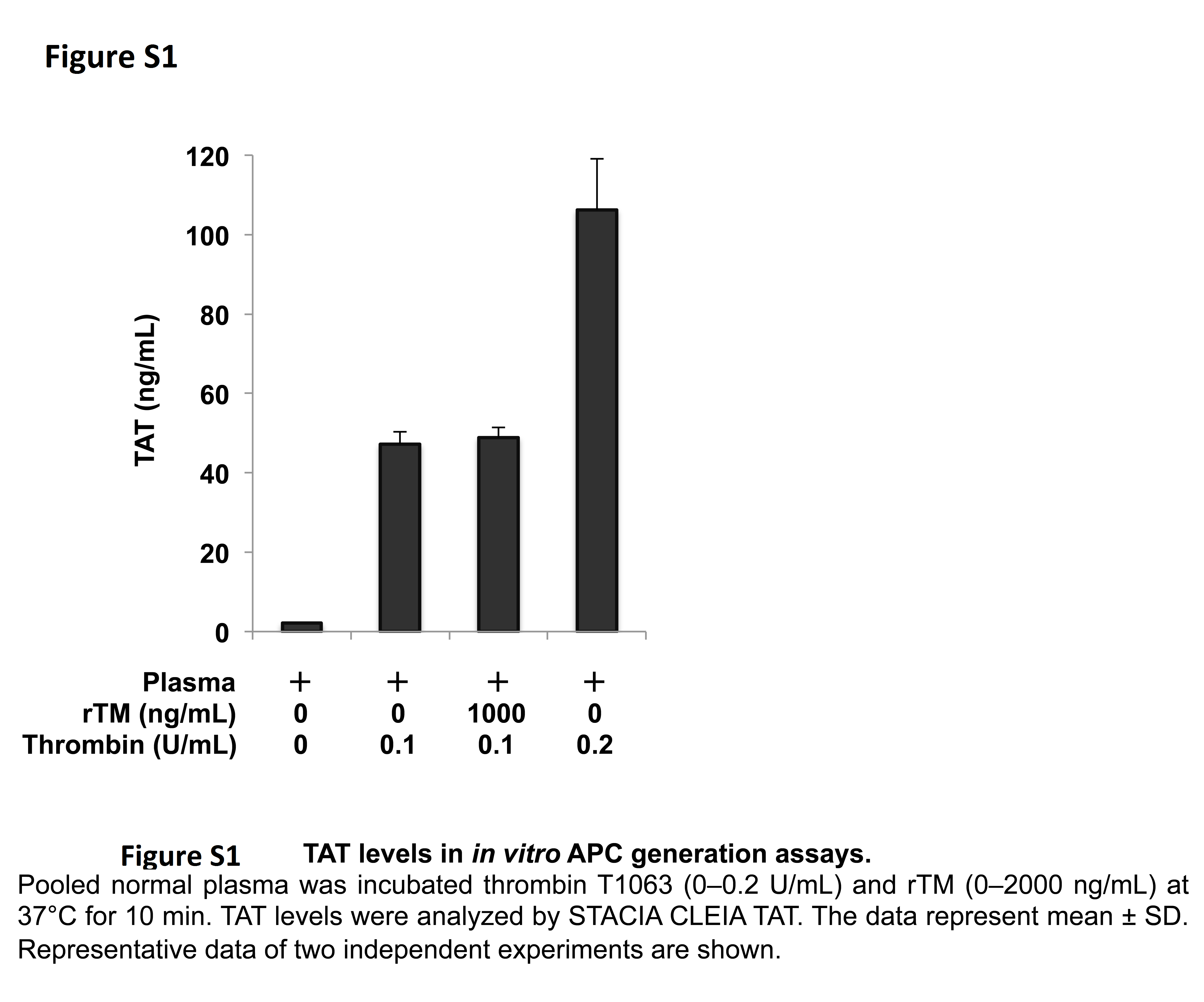

Supplement: Supplementary file 1 — Figure S1. TAT levels in in vitro APC generation assays. Pooled normal plasma was incubated with thrombin T1063 (0–0.2 U/mL) and rTM (0–2000 ng/mL) at 37 °C for 10 min. TAT levels were analyzed by STACIA CLEIA TAT. The data represent mean ± SD. Representative data of two independent experiments are shown. (TIF 547 kb) [file 12959_2018_178_MOESM1_ESM.tif]

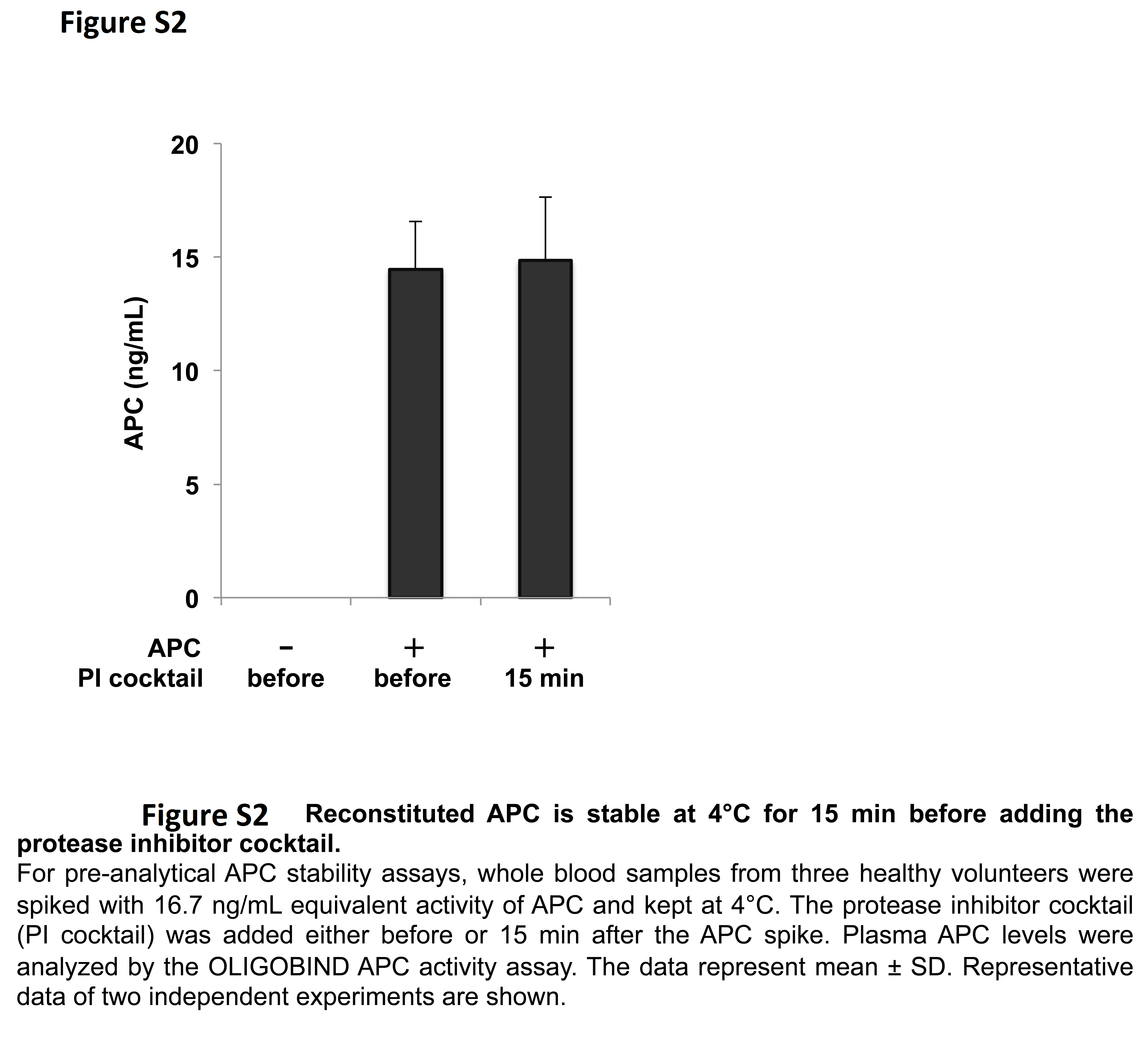

Supplement: Supplementary file 2 — Figure S2. Reconstituted APC is stable at 4 °C for 15 min before adding the protease inhibitor cocktail. For pre-analytical APC stability assays, whole blood samples from three healthy volunteers were spiked with 16.7 ng/mL equivalent activity of APC and kept at 4 °C. The protease inhibitor cocktail (PI cocktail) was added either before or 15 min after the APC spike. Plasma APC levels were analyzed by the OLIGOBIND APC activity assay. The data represent mean ± SD. Representative data of two independent experiments are shown. (TIF 640 kb) [file 12959_2018_178_MOESM2_ESM.tif]
